# Retraction: Interferon characterization associates with asthma and is a potential biomarker of predictive diagnosis

**DOI:** 10.1042/BSR-20204210_RET

**Published:** 2021-05-04

**Keywords:** asthma, biomarker, blood, diagnosis, IFN

This article is being retracted from *Bioscience Reports* at the request of the Editorial Board following receipt of a notification from a reader, alerting the Editorial Office to significant similarity with another article, published by different authors (10.1089/dna.2020.5543) which is cited as reference [1] in the *Bioscience Reports* paper.

Both papers examined the same datasets from the Gene Expression Omnibus however, looked at different genes. Text from the Introduction, Materials and Methods, Results and Discussion overlaps with the other publication with minor changes to the language, but keeping the same general structure and meaning.

The authors have been contacted in regard to the retraction, and agree with the Journal's decision to retract the article.

